# Cili (*Rosa roxburghii* Tratt.) as a Functional Food and Medicinal Resource: Current Advances and Future Directions

**DOI:** 10.3390/cimb48030249

**Published:** 2026-02-26

**Authors:** Jiaqiang Zhang, Xiang Li, Qi Wang, Fei Wang, Bin Deng, Yongbin Han, Weifeng Li, Rongchang Yang

**Affiliations:** 1 Zhejiang Institute of Landscape Plants and Flowers, Zhejiang Academy of Agricultural Sciences, Hangzhou 311251, China; zhangqiang414@126.com; 2 Institute of Animal Nutrition and Feed Sciences, College of Animal Sciences, Zhejiang University, Hangzhou 310058, China; 979286096@163.com (X.L.); 13430230952@163.com (Q.W.); 22017007@zju.edu.cn (F.W.); 3 Department of Light Industry and Chemical Engineering, Guizhou Light Industry Polytechnic University, Guiyang 550005, China; dengyun85@hotmail.com; 4 College of Food Science & Technology, Nanjing Agricultural University, Nanjing 211816, China; hanyongbin@njau.edu.cn; 5 School of Agricultural Sciences, Murdoch University, Murdoch, WA 6150, Australia

**Keywords:** Cili, phytochemistry, bioactive compounds, vitamin C, superoxide dismutase, antioxidation, inflammation, metabolic homeostasis, omics-driven application

## Abstract

*Rosa roxburghii* Tratt., commonly known as Cili, is an emerging functional fruit native to southwestern China, characterized by extraordinarily high vitamin C content, robust superoxide dismutase activity, and a rich diversity of bioactive compounds. This review summarizes recent advances in its phytochemistry, molecular bioactivity, and omics-driven applications, with a focus on integrating evidence from genomic, transcriptomic, and metabolomic analyses. Multi-omics analyses reveal the coordinated regulation of ascorbate and secondary metabolite accumulation via key pathways and transcription factors. Mechanistically, bioactive compounds from Cili comprising ascorbate derivatives, polyphenols, flavonoids, polysaccharides, triterpenoids, and sterols, act synergistically. They also exhibit antioxidant, anti-inflammatory, gastrointestinal, hepatoprotective, cardiovascular protective, anti-obesity, anti-diabetic, metabolic regulatory, anti-cancer, and neuroprotective effects by modulating core metabolic and stress-response signaling pathways. Recent advances in functional food processing have further elevated its nutritional and industrial value, highlighting its potential as a sustainable nutraceutical resource.

## 1. Introduction

*Rosa roxburghii* Tratt., commonly known as Cili or chestnut rose, is a perennial shrub belonging to the family Rosaceae that is native to the karst regions of southwestern China. It is renowned for its exceptionally high vitamin C content, elevated superoxide dismutase (SOD) activity, and a wide spectrum of bioactive phytochemicals, including phenolic acids, flavonoids, triterpenoids, and polysaccharides, which collectively contribute to its strong antioxidant and therapeutic potential [1,2,3]. The fruit of Cili has long been used as both food and medicine, especially for its health-promoting and anti-aging properties and has recently emerged as a model species for nutritional genomics and metabolomics research within the Rosaceae [4].

Recent multi-omics studies have elucidated the molecular mechanisms underlying the abundant accumulation of bioactive compounds in Cili. Transcriptomic and metabolomic profiling during fruit development has revealed gene–metabolite networks associated with vitamin C biosynthesis, highlighting the involvement of key structural genes such as *RrGGP* and *RrGalUR*, which are regulated by transcription factors including *RrHY5H*, *RrZIP9*, *RrZIP6*, and *RrWRKY4* [5]. Similarly, integrative metabolomic–transcriptomic approaches have identified regulatory pathways for amino acids, phenolic acids, flavonoids, and triterpenoids, highlighting the pivotal roles of *RrMYB* transcription factors and members of CYP/UGT gene families in secondary metabolism [2,6,7]. Together, these findings provide insight into the genetic regulation of phytochemical biosynthesis and the ecological adaptability of Cili in its native karst environments.

Beyond its phytochemical richness, Cili exhibits considerable tolerance to abiotic stresses and can thrive in calcareous and drought-prone soils. This resilience has been associated with the co-expression of ion-transport and antioxidant defense genes, suggesting strong adaptive potential relevant to stress physiology and horticultural improvement [1]. Collectively, these characteristics position Cili as an underutilized yet promising functional crop species with significant nutritional, medicinal, and industrial applications.

While several previous reviews have summarized the phytochemical composition and general health benefits of Cili, most have focused primarily on descriptive accounts of nutrient content or isolated pharmacological effects. However, the present review provides an integrated, application-oriented synthesis that connects multi-omics advances, including genomics, transcriptomics, metabolomics, and network pharmacology, with mechanistic interpretations of bioactivity and translational utilization. Specifically, this review emphasizes gene–metabolite regulatory networks governing phytochemical biosynthesis, system-level signaling pathways underlying biological effects, and omics-guided strategies for functional food development, nutraceutical innovation, and circular utilization of processing by-products. By bridging molecular mechanisms with industrial and nutritional applications, this review aims to provide a comprehensive framework supporting the future development of Cili as a functional crop and medicinal food resource.

## 2. Morphology and Genetic Characteristics of Cili

### 2.1. Morphology

Cili is recognized for its distinct morphological features, including compound serrated leaves, solitary pink flowers, and fruits adorned with a unique burr-like surface comprising numerous spines. The species exhibits substantial phenotypic diversity, reflecting adaptation to heterogeneous ecological niches within the karst environment. Morphological studies have demonstrated that fruit prickles are multicellular appendages that originate from the ground meristem during early fruit development, which subsequently lignify to form the hardened spiny surface at maturity [7]. Histological analyses further indicate that prickle primordia formation is regulated by coordinated cell division and expansion, resulting in pronounced polymorphism across genotypes and developmental stages.

At the molecular level, Cili exhibits trichome and prickle formation mechanisms comparable to those of Arabidopsis thaliana. The GLABROUS1 (*RrGL1*) gene, a homolog of *AtGL1*, regulates trichome initiation through interaction with basic helix-loop-helix transcription factors such as *GL3* and *EGL3* [8]. Functional complementation experiments have demonstrated that *RrGL1* can restore trichome formation in Arabidopsis *gl1* mutants, supporting its conserved regulatory function in trichome development. Similarly, Cili TG1, a WD40-repeat protein, forms an MBW (MYB-bHLH-WD40) complex with *RrGL1* and *RrEGL3*, thereby activating epidermal cell differentiation and promoting prickle development [7].

Fruit development in Cili is intricately regulated by phytohormones, particularly gibberellins (GAs). Transcriptomic analysis has revealed that exogenous GA_3_ treatment stimulates fruit enlargement, induces seed abortion, and enhances ascorbic acid accumulation. These morphological and biochemical changes are mediated by the transcriptional reprogramming of carbon metabolism, glycolysis, and phytohormone signaling genes [9]. Ascorbic acid biosynthesis, which increases sharply during fruit ripening, is strongly associated with the L-galactose pathway and enhanced activity of dehydroascorbate reductase (DHAR), a key enzyme maintaining redox homeostasis [10]. Morphological damage induced by biotic stress also influences fruit physiology. For example, Grapholita molesta (oriental fruit moth) feeding on Cili fruit leads to substantial biochemical alterations, including the reduced superoxide dismutase and vitamin C levels and the increased bitterness, as confirmed by metabolomic analyses [11].

Overall, the morphology of Cili reflects the complex genetic regulation of epidermal cell differentiation, hormone-dependent fruit growth, and defense adaptations—traits that underpin its ecological resilience and economic value as a functional fruit crop.

### 2.2. Genetic Characteristics

The increasing availability of transcriptomic and genomic resources has substantially advanced our understanding of the genetic underpinnings of Cili development and secondary metabolism. De novo transcriptome assembly from multiple tissues has identified over 63,000 unigenes, including 163 MYB transcription factor genes, many of which are tissue-specific and involved in regulating phenylpropanoid biosynthesis, fruit pigmentation, and prickle formation [7]. Comparative expression analyses have confirmed that R2R3-MYB and atypical MYB-like genes play critical roles in flavonoid metabolism and epidermal differentiation, underpinning the morphological and phytochemical diversity of the species.

Light-regulated ascorbate biosynthesis represents another major metabolic innovation in Cili. The transcription factor Cycling Dof Factor 3 (*RrCDF3*) directly activates GDP-L-galactose phosphorylase (*RrGGP2*), a rate-limiting enzyme in the L-galactose pathway, in a light-dependent manner [12]. Functional assays have demonstrated that *RrCDF3* interacts with the photoreceptor-associated transcription factor *RrHY5*, forming a regulatory cascade that enhances vitamin C accumulation in fruit. This elucidated *RrHY5–RrCDF3–RrGGP2* module offers a mechanistic explanation for the exceptionally high ascorbate content characteristic of Cili, distinguishing it from other members of the Rosaceae family.

Genomic resources have further supported taxonomic and evolutionary studies. The plastome of Cili has been annotated using the PGA (Plastid Genome Annotator) tool, which facilitates the precise identification of coding regions, intron losses, and genome rearrangements—features that are instrumental for robust phylogenetic analysis within *Rosa* [13]. Moreover, 33 polyphenolic compounds with antioxidant and anti-aging potential, linked to the insulin/IGF-1 signaling pathway have been identified by network pharmacology and metabolomic profiling of Cili seeds [14]. These findings underscore the intricate interconnection between Cili’s genomic architecture and its metabolomic profile, thereby establishing a solid foundation for synergistic evolutionary and functional research.

**Figure 1:**
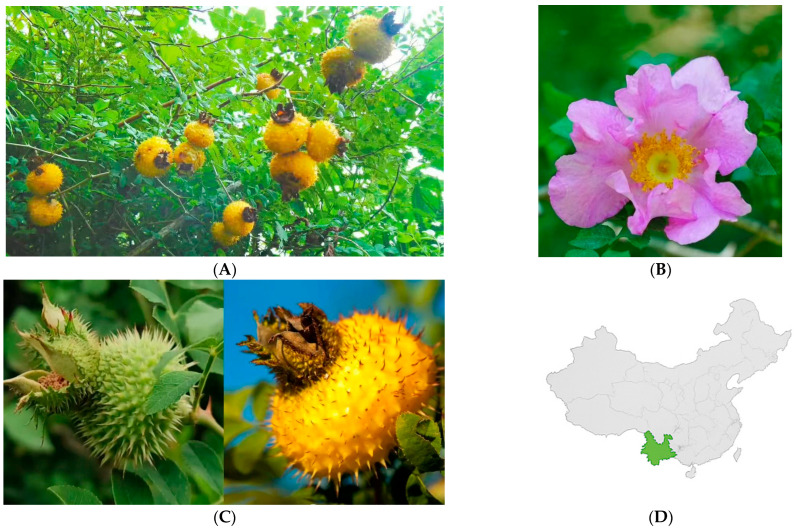
Morphological and Ecological Characteristics of Cili (Rosa roxburghii Tratt.). (**A**) Whole shrub and fruiting branch; (**B**) Floral morphology; (**C**) Distinctive spiny fruit morphology characteristic of mature Cili; (**D**) Geographic distribution of Cili in southwestern China, primary in Guizhou, Yunnan, and Guangxi provinces.

Collectively, advances in transcriptomic, metabolomic, and genomic studies have illuminated the molecular basis of the species’ distinctive traits, including its rich antioxidant profile, high vitamin C content, and adaptive morphological features, establishing Cili as a valuable model for studying fruit physiology and metabolic evolution in the Rosaceae family (Figure 1).

## 3. Phytochemical Composition and Functional Significance

The exceptional health-promoting capacity of Cili is largely attributed to its complex and diverse phytochemical profile. The fruit contains high concentrations of vitamin C, SOD, polyphenols, flavonoids, polysaccharides, triterpenoids, sterols, organic acids, and dietary fibers, each contributing to its strong antioxidant and pharmacological effects. Furthermore, integrative approaches combining biochemical, metabolomic, and transcriptomic analyses have begun to unravel the molecular basis governing the biosynthesis and synergistic interactions of these phytochemicals, thereby providing a scientific foundation for their observed health benefits [9,14,15].

### 3.1. Vitamin C, Superoxide Dismutase, and Antioxidant Compounds

Cili is recognized as one of the richest natural sources of vitamin C among edible fruits, often referred to as the “King of Vitamin C”. The concentration of L-ascorbic acid can reach more than 1700 mg per 100 g fresh weight, far exceeding that in most commonly consumed citrus fruits. The fruit’s antioxidant system is strengthened by enzymatic components such as SOD, catalase (CAT), and peroxidase, working in concert with non-enzymatic antioxidants like flavonoids and polyphenols [15].

Recent transcriptomic evidence has revealed that the *RrGGP2* gene plays a crucial role in ascorbate biosynthesis, while the transcription factor *RrNAC56* regulates *RrGGP2* expression under cold stress, thereby promoting vitamin C accumulation [16]. Further analysis of *RrGGP2* promoter elements identified multiple stress-responsive cis-elements and abscisic acid (ABA) regulatory sites. These findings confirm that the maintenance of exceptionally high ascorbate levels in Cili is achieved through a sophisticated regulatory network that integrates both developmental cues and environmental signals. This remarkable vitamin C content acts in concert with the fruit’s abundant phenolic compounds and polysaccharides, resulting in a synergistic enhancement of its overall antioxidant capacity [14].

### 3.2. Polyphenols and Flavonoids

Polyphenols and flavonoids constitute the principal classes of secondary metabolites responsible for the potent antioxidant, anti-inflammatory, and metabolic health-promoting properties of Cili. Comprehensive LC-MS-based metabolomic profiling has led to the identification of over 500 distinct phenolic compounds, with representative constituents including quercetin, catechin, kaempferol, rutin, and gallic acid derivatives [2,17]. These compounds are synthesized via the phenylpropanoid and flavonoid biosynthetic pathways, which are regulated by transcription factors such as *RrMYB*, *RrWRKY*, and *RrTCP* [9].

Beyond their direct antioxidant activity, flavonoids exert a dual role as antioxidants and signaling molecules, regulating key cellular pathways such as PI3K–Akt, PPAR, and NF-κB, thereby maintaining redox homeostasis and inhibiting inflammatory mediators [18]. In addition, polyphenolic components contribute to the characteristic aroma and flavor of the fruit, with benzaldehyde, furfuryl acetate, and ethyl hexanoate identified as major aroma-active volatile compounds [17,19]. The co-accumulation of phenolic acids and flavonoids acts synergistically to strengthen the antioxidant and cytoprotective properties of the fruit [15].

### 3.3. Polysaccharides

Polysaccharides represent another key class of bioactive macromolecules in Cili, contributing to antioxidant, immunomodulatory, and antitumor activities. Structural characterization has revealed that these polysaccharides are mainly composed of arabinose, galactose, glucose, and rhamnose residues, forming complex backbones of rhamnogalacturonan-I and homogalacturonan [20]. Among them, the RP1 and RP3 fractions exhibit distinct structural configurations, with RP1 rich in arabinogalactan side chains that enhance free radical-scavenging capacity and apoptosis-inducing effects in cancer cells.

Extraction methods significantly influence the yield and structural integrity of Cili polysaccharides. Ultrasound-assisted enzymatic extraction yields a higher polysaccharide recovery while preserving their bioactivity, outperforming traditional extraction methods [20]. Regarding their mechanisms of action, Cili polysaccharides exert antioxidant effects by activating endogenous cellular antioxidant enzymes and suppressing generation of reactive oxygen species (ROS) [15]. Moreover, emerging evidence suggests that these polysaccharides are involved in modulating lipid metabolism and gut microbiota composition, thereby establishing a connection to broader metabolic health benefits [21].

### 3.4. Triterpenoids and Sterols

Triterpenoids represent a signature group of bioactive metabolites in Cili, exhibiting diverse biological activities, including anticancer, hepatoprotective, anti-inflammatory, and lipid-regulatory effects. Several pentacyclic triterpenoids, such as ursolic acid, oleanolic acid, and arjunic acid, have been isolated from the fruits and roots of this species [22]. Among these, the saponin kaji-ichigoside F1 has been identified as a key bioactive compound that modulates the PPAR-γ, Nrf2, and NF-κB signaling pathways, thereby attenuating oxidative stress and inflammatory responses [23].

Proteomic analyses further demonstrate that triterpenoid-rich Cili extracts modulate lipid metabolism by regulating key hepatic enzymes and transport proteins, thereby reducing triglyceride and cholesterol levels in hyperlipidemic mice [24]. The coordinated accumulation of triterpenoids, sterols, and ascorbic acid points to a metabolic crosstalk between secondary metabolism and the antioxidant defense system. This coordinated phytochemical profile aligns with the species’ evolutionary adaptation to the oxidative stressors inherent to its native karst habitats.

**Figure 2:**
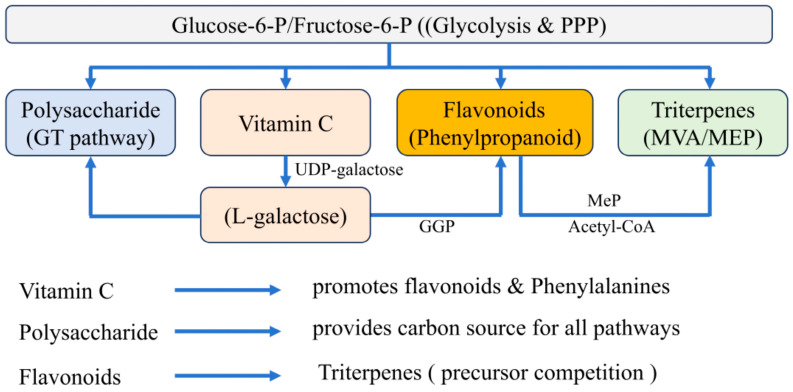
Biosynthetic Pathways of Bioactive Compounds in Cili (*Rosa roxburghii* Tratt.)

An overview of the major biosynthetic pathways and representative metabolites of Cili is shown in Figure 2.

### 3.5. Other Phytochemicals and Synergistic Interactions

Beyond the major classes of specialized metabolites, Cili fruit contains abundant organic acids (e.g., citric, malic, and tartaric acids), essential amino acids, and dietary fiber that contribute to its organoleptic, nutritional, and functional properties [11]. These organic acids not only enhance flavor profiles but also promote mineral absorption, while soluble dietary fiber modulates gut microbiota homeostasis. Moreover, polysaccharides, polyphenols, flavonoids, and triterpenoids exert synergistic biological effects that are more potent than those of any single compound [14]. This phytochemical synergy underlies the superior antioxidant and metabolic benefits of whole-fruit extracts, emphasizing the importance of preserving the natural phytochemical integrity during product development and processing.

## 4. Molecular Mechanisms of Bioactive Compounds in Cili

The extensive and diverse pharmacological potential of Cili arises from the synergistic interactions among its key bioactive constituents, including vitamins, flavonoids, triterpenoids, polysaccharides, and polyphenols. These compounds collectively modulate a wide range of critical cellular pathways involved in oxidative stress, inflammatory responses, metabolic regulation, programmed cell death (apoptosis), and immune homeostasis. Recent metabolomic, transcriptomic, and network pharmacology studies have validated that Cili alleviates oxidative stress-related and metabolic disorders by regulating the PI3K–AKT, AMPK, PPAR, Nrf2/HO-1, MAPK, and NF-κB signaling cascades, thereby improving redox homeostasis and overall systemic health [25,26,27].

### 4.1. Antioxidant and Anti-Inflammatory Activities

Both in vitro and in vivo studies have consistently demonstrated that phenolic-, triterpenoid-, and polysaccharide-rich fractions of Cili effectively scavenge reactive oxygen species (ROS) and enhance endogenous antioxidant defenses, including superoxide dismutase (SOD), catalase (CAT), and glutathione peroxidase (GPx) [15,26,28].

Mechanistically, these antioxidant effects converge on the activation of redox-sensitive transcriptional regulators, particularly the Nrf2/HO-1 pathway, which is potently stimulated by enzymatically extracted polysaccharides and further reinforced by flavonoid- and triterpenoid-mediated signaling [27,29].

In parallel, flavonoids and triterpenoids derived from Cili exert potent anti-inflammatory effects by downregulating the expression of pro-inflammatory cytokines (TNF-α, IL-6, and IL-1β) and inhibiting activation of *NLRP3* inflammasome activation, thereby attenuating inflammation driven by oxidative damage [27].

Taken together, these findings indicate that the antioxidant and anti-inflammatory activities of Cili are not compound-specific but arise from a coordinated phytochemical network that simultaneously enhances antioxidant capacity and suppresses pro-inflammatory signaling. This notion is further supported by the dual antibacterial and antioxidant activities observed in Cili-derived selenium nanoparticles [30].

### 4.2. Gastrointestinal and Hepatoprotective Effects

Cili exerts remarkable beneficial effects on gut microbiota regulation and intestinal health mainly by modulating the gut-liver axis and maintaining intestinal homeostasis [31,32,33,34,35,36]. Polyphenols, polysaccharides, and fermented Cili products enhance endogenous antioxidant enzyme activities, alleviate oxidative stress, suppress inflammatory signaling, restore gut microbial balance, and maintain intestinal barrier integrity, thus reducing hepatic oxidative injury induced by intestinal disorders [32,33]. Mechanistically, the regulatory effects of Cili are closely associated with the modulation of NF-κB signaling, bile acid metabolism, and microbial-derived metabolites, which jointly underpin its gastrointestinal protective function [32,33,34,35]. Polyphenol-rich Cili extracts can effectively ameliorate colitis-associated liver inflammation by inhibiting pro-inflammatory lipid mediators and protecting the intestinal mucosal structure [34,35]. Fermented Cili juice further improves bile acid metabolism and optimizes the composition and structure of the gut microbiota, contributing to enhanced intestinal antioxidant capacity and mucosal stability [31]. Furthermore, selenium-enriched Cili polysaccharides help restore microbial homeostasis and protect against heavy-metal-induced organ damage, providing an additional mechanism for intestinal and systemic protection [36]. Taken together, Cili effectively modulates gut microbiota and improves intestinal health through synergistic regulation of intestinal barrier function, oxidative balance, microbial composition, and gut-liver crosstalk.

### 4.3. Immune Regulation

Cili exerts significant immunomodulatory effects through the synergistic action of its key bioactive components, including polysaccharides, flavonoids, and selenium-enriched derivatives [6,35,36,37]. Cili polysaccharides, the primary immunomodulatory constituents, enhance the activity of innate immune cells (macrophages, NK cells), promote splenic lymphocyte proliferation and differentiation, improve immune organ function, and regulate Th1/Th2 cell balance to alleviate immune disorders [3,4,7]. Cili flavonoids maintain immune homeostasis by inhibiting ROS production, blocking NLRP3 inflammasome and NF-κB signaling activation, and reducing the release of pro-inflammatory mediators [2,4]. Additionally, selenium-enriched Cili polysaccharides restore gut microbiota balance and enhance resistance to external damage, while Cili-rich vitamin C and SOD synergistically scavenge free radicals to protect immune cells [5,6,8,36]. Collectively, these components enable Cili to maintain systemic immune stability as a valuable natural immunonutrient, acting via multi-target regulation rather than nonspecific immune stimulation [37].

### 4.4. Anti-Obesity, Anti-Diabetic, and Metabolic Regulatory Effects

Cili exerts comprehensive regulatory effects on anti-obesity, anti-diabetic, and systemic metabolism through the coordinated modulation of multiple signaling pathways, mitochondrial function, epigenetic mechanisms, and gut microbiota. For anti-diabetic and metabolic regulation, Cili bioactives (including polysaccharides, polyphenols, and triterpenoids) act as integrative regulators of glucose and lipid metabolism by activating AMPK-, PPAR-, and PI3K-AKT-dependent signaling pathways, which enhance fatty acid oxidation, suppress hepatic lipogenesis, improve insulin sensitivity, and attenuate oxidative stress [18,31]; these benefits are further reinforced by modulating gut microbiota composition (enriching short-chain fatty-acid-producing genera and regulating bile acid metabolism) and microbial metabolites, as observed in fermented Cili juice and vinegar formulations that promote hepatic lipid turnover and systemic metabolic homeostasis [2,21,38]. Meanwhile, the anti-obesity effects of Cili arise from integrated regulation of mitochondrial function, lipid metabolism, and epigenetic control of metabolic genes: Cili extracts alleviate lipid accumulation by activating PI3K–AKT and PPAR signaling while enhancing mitochondrial oxidative capacity [18], fermentation-derived flavonoids regulate adipogenesis via DNMT3a/SIRT1-mediated epigenetic modulation to reinforce long-term metabolic reprogramming [39], and these transcriptional and epigenetic effects converge with AMPK-SIRT1 signaling to restore energy homeostasis and metabolic flexibility under high-fat dietary conditions [21]. Collectively, these multi-target regulatory mechanisms enable Cili to simultaneously exert anti-obesity, anti-diabetic, and systemic metabolic improvement effects.

### 4.5. Cardiovascular Protective Effects

Cardiovascular protection conferred by Cili is primarily mediated through enhancement of endothelial function and redox balance. Polyphenols and triterpenoids activate the PI3K–AKT–eNOS signaling cascade, promoting nitric oxide bioavailability and alleviating vascular oxidative stress [27]. In parallel, Cili-derived extracts regulate lipid metabolism and strengthen antioxidant defenses, thereby improving vascular homeostasis and lowering atherogenic risk [24,31]. Comparative studies across *Rosa* species further suggest that flavonol glycosides with conserved antioxidant activity contribute to an evolutionarily conserved cardiovascular protective mechanism within the Rosaceae family [40].

### 4.6. Anti-Cancer Effects

Although direct clinical evidence remains limited, mechanistic and cellular studies consistently demonstrate that Cili bioactives exert anticancer potential through ROS-mediated apoptotic signaling, cell cycle regulation, and genomic protection. Polysaccharides suppress cancer cell proliferation and induce apoptosis via MAPK-, STAT-, and p53-dependent pathways in ovarian, prostate, and hepatic carcinoma models [41,42,43]. Triterpenoids from Cili, such as euscaphic acid, further trigger mitochondrial apoptosis through ROS/MAPK signaling in colorectal cancer cells [44]. In addition, Cili flavonoids demonstrate radioprotective effects by preventing oxidative DNA damage, supporting their complementary role in maintaining genomic stability [45]. Network pharmacology analyses have identified quercetin, kaempferol, and ellagic acid as key multi-target phytochemicals from Cili, which are predicted to interact with tumor-associated proteins, including TP53 [46].

### 4.7. Neuroprotective and Other Pharmacological Activities

Neuroprotective effects of Cili are predominantly mediated through the suppression of neuroinflammation and reinforcement of neurotrophic signaling pathways. The triterpenoid saponin kaji-ichigoside F1 activates the PPAR-γ/CX3CR1/Nrf2 axis while concurrently inhibiting NF-κB/NLRP3 inflammasome activation, thereby alleviating neuroinflammatory injury [47]. Additional studies have demonstrated that Cili extracts modulate BDNF/Akt/mTOR signaling to enhance neuronal survival and neurotransmitter balance under excitotoxic and stress-related conditions [48]. Furthermore, in vivo evidence supports the safety of Cili in the central nervous system as well as its adaptogenic properties, including protection against ethanol-induced psychomotor impairment [49].

### 4.8. Integrative Summary

**Figure 3:**
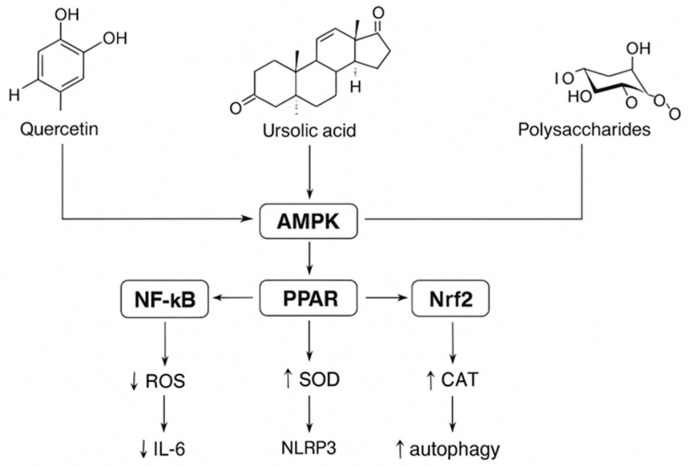
Mechanistic Overview of the Antioxidant, Anti-inflammatory, and Metabolic Regulation Effects of Cili (*Rosa roxburghii* Tratt.). (Figure was originally generated based on [46,48].)

Accumulating evidence substantiates that Cili elicits multi-target pharmacological effects by integrating antioxidant, metabolic, and immunomodulatory signaling pathways. The main molecular mechanisms involve the activation of Nrf2/HO-1, AMPK, and PPAR pathways; the inhibition of PI3K–AKT–NF-κB/NLRP3 cascades; and the restoration of microbial and metabolic homeostasis. These coordinated processes, validated by transcriptomic, metabolomic, and cellular studies, demonstrate that the beneficial effects of Cili stem from synergistic interactions among its diverse phytochemical constituents, rather than from individual compounds. Furthermore, comparative phytochemical and genomic analyses within the Rosaceae family have revealed evolutionarily conserved redox and metabolic regulatory networks, establishing Cili as a promising model for the development of plant-derived functional foods and therapeutic agents. The key biological activities of Cili supported by experimental evidence are summarized in Table 1 and Figure 3.

## 5. Applications and Prospects of Cili in Functional Foods and Nutraceuticals

**Figure 4:**
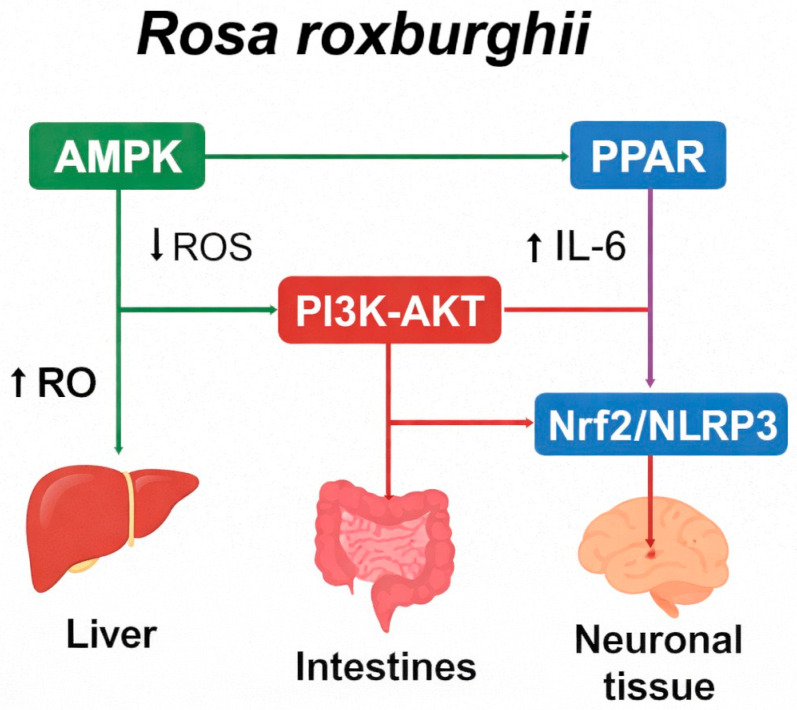
Integrated Molecular Pathways of Cili (*Rosa roxburghii* Tratt.) bioactivity. (Figure was originally generated based on [51]).

The expanding understanding of Cili genomics, metabolomics, and molecular bioactivity has positioned this fruit as a high-value resource for the development of functional foods and nutraceuticals. Integrative omics approaches have elucidated the complex synergistic interactions among vitamins, flavonoids, polysaccharides, and triterpenoids that underpin the fruit’s health-promoting properties [2]. With advances in metabolic profiling, the specific biochemical pathways that contribute to antioxidant capacity and flavor characteristics are being deciphered, providing guidance for precision processing and product formulation. This section outlines the translation of these scientific insights into practical applications, covering functional foods, nutraceutical development, and sustainable circular utilization (Figure 4).

### 5.1. Functional Food

Cili fruit extracts have been effectively applied in beverages, functional snacks, and fermented health drinks, where their bioactive compounds contribute to enhanced antioxidant intake and improved metabolic health. This practical application is guided by fundamental research: multi-omics analyses have delineated key gene–metabolite networks that regulate the synthesis of vitamin C and flavonoids during fruit maturation [2]. Fermentation strategies have emerged as particularly effective tools for improving sensory attributes and bioavailability. For example, tandem fermentation involving edible mushrooms and *Lactobacillus plantarum* markedly increased contents of γ-aminobutyric acid (GABA), polysaccharides, and vitamin C in Cili seed beverages [52].

Further optimization of Cili pomace fermentation using both mono- and mixed-culture approaches has been shown to improve the phytochemical profile and boost the antioxidant potential of the resulting fermented substrates [53]. These findings demonstrate that microbial co-fermentation not only upgrades functional quality but also valorizes by-products that would otherwise be discarded. Similarly, enzyme-assisted extraction and targeted heating techniques have been employed to improve the solubility and retention of polysaccharides and phenolic acids in processed foods [20]. Whole-fruit formulations, which preserve the natural matrix of vitamin C, SOD, flavonoids, and triterpenoids, consistently outperform purified compounds in terms of antioxidant capacity, reflecting synergistic effects among Cili’s phytochemicals and enzymes.

From a technological perspective, the incorporation of Cili into functional foods requires careful control of processing conditions to preserve labile bioactive compounds, particularly vitamin C, polyphenols, and redox-active enzymes. Fermentation has emerged as an effective stabilization strategy, as microbial metabolism mitigates oxidative degradation while simultaneously improving sensory properties and bioavailability [52,53]. Compared with conventional high-temperature processing, enzyme-assisted extraction and mild thermal treatment further enhance polysaccharide solubility and phenolic retention, thereby maintaining antioxidant activity [20]. Importantly, whole-fruit and matrix-preserving formulations consistently exhibit higher antioxidant capacity than isolated compounds, underscoring the protective role of the natural phytochemical matrix during processing and storage [2]. These findings highlight that appropriate processing and stabilization strategies are essential for translating Cili bioactivity into reproducible, high-quality functional food products.

### 5.2. Nutraceutical Development

Beyond food applications, Cili bioactives are increasingly incorporated into nutraceutical formulations for antioxidant, hepatoprotective, and lipid-regulatory purposes. In a controlled animal model, fermented Cili juice significantly alleviated high-fat-diet-induced hyperlipidemia by modulating gut microbiota composition and short-chain fatty acid production [31]. Similar findings were reported in type 2 diabetes mice, where *Lactobacillus paracasei*-fermented Cili juice restored oxidative balance and corrected microbial dysbiosis [50].

Cili fruit extracts also confer notable gastrointestinal and hepatic protection. Supporting this, in a murine model of dextran sulfate sodium (DSS)-induced colitis, Cili polyphenols were shown to regulate IL-17-associated immune pathways and strengthen intestinal mucosal barrier function [54]. Furthermore, network pharmacology and cellular assays demonstrate that Cili-derived triterpenoids and flavonoids activate PI3K–Akt and PPAR signaling pathways, thereby enhancing lipid oxidation and energy metabolism [18]. Collectively, these studies provide mechanistic evidence for Cili’s multi-target bioactivity—linking antioxidant defense, microbial modulation, and metabolic regulation to its gastrointestinal, hepatic, and metabolic protective effects.

### 5.3. By-Products and Circular Utilization

Valorization of Cili by-products including pomace, peel, and seeds offers promising opportunities for sustainable bioprocessing. These residual fractions contain high concentrations of dietary fiber, flavonoids, and triterpenoids that retain substantial antioxidant potential. Lactic acid bacteria isolated from naturally ensiled pomace exhibit strong antioxidant and preservative capacities, suggesting their utility in probiotic formulations [55].

In another study, Ganoderma-fermented Cili pomace was incorporated into wheat noodles, enhancing their antioxidant capacity and modulating gut microbial composition [56]. Industrial-scale applications are also gaining momentum. For example, pectin and polysaccharides derived from Cili pomace have been utilized to fabricate biodegradable active films for food packaging, which effectively extend shelf life [57]. Concurrently, residual Cili biomass has been converted into hard carbon anodes for sodium-ion batteries, illustrating an innovative biorefinery approach [58]. Such circular utilization strategies exemplify how waste reduction can coincide with the creation of functional and economic value.

### 5.4. Multi-Omics-Guided Breeding and Metabolic Engineering

Recent multi-omics studies have clarified the genetic and transcriptional regulation of vitamin C, flavonoid, and triterpenoid biosynthesis in Cili. Structural analysis of the *RrGGP2* promoter identified stress-inducible cis-elements that mediate ascorbate synthesis via the transcription factor *RrNAC56* [16]. Complementary work has identified the *RrHY5*–*RrCDF3*–*RrGGP2* regulatory module as a major determinant of fruit ascorbate accumulation [5]. Integration of metabolite–gene networks have further uncovered key members of the MYB, WRKY, and P450 family that are responsible for the tissue-specific biosynthesis of flavonoids and triterpenoids [2].

These advances provide essential molecular targets for marker-assisted breeding, while synthetic biology approaches are being explored to transfer high-efficiency biosynthetic genes into microbial systems for the sustainable production of target metabolites. Such strategies parallel similar developments in other Rosaceae species, where flavonol acylation and glycosylation pathways have been leveraged to breed antioxidant-rich cultivars [40].

### 5.5. Safety Considerations, Dosage, and Limitations

Cili has a long history of dietary and medicinal use in Southwest China, where the fruit is traditionally consumed fresh or processed into juices, wines, vinegars, and fermented products, with no reports of severe adverse effects associated with customary intake [1,22,37]. This long-standing consumption provides important empirical support for its general safety as a functional food.

Available preclinical studies indicate that aqueous extracts, ethanolic extracts, polysaccharide fractions, polyphenol-rich extracts, and fermented products of Cili exhibit low acute and sub-chronic toxicity in animal models when administered orally within commonly tested dosage ranges [2,31,34,47,49,54]. In studies evaluating antioxidant, anti-inflammatory, metabolic, hepatoprotective, and gut-protective effects, effective doses typically ranged from 50 to 500 mg·kg^−1^·day^−1^, with no significant adverse effects observed on body weight, organ indices, or serum biochemical parameters [2,6,15,24,31].

However, dosage regimens and extract compositions vary substantially among studies due to differences in plant material origin, extraction methods, fermentation processes, and phytochemical profiles, underscoring the need for standardized preparations and marker-based quality control [1,20,25,26,29]. The increasing use of enzyme-assisted extraction, fermentation, and metabolomics-guided characterization highlights ongoing efforts to improve reproducibility and safety consistency [25,26,29].

Despite promising preclinical evidence, systematic human safety data remain limited, and well-designed clinical studies assessing long-term intake, dose–response relationships, and potential interactions with pharmaceuticals are still lacking. Given the reported effects of Cili extracts on oxidative stress, glucose and lipid metabolism, immune regulation, and gut microbiota composition, potential interactions—particularly in individuals receiving antioxidant supplementation or metabolic therapies—should be carefully evaluated in future studies [2,34,35,54] (Table 2).

### 5.6. Future Prospects and Challenges

Despite rapid progress, several challenges remain in ensuring product standardization and bioactive stability of Cili-based products. Genetic diversity, environmental conditions, and processing variables all influence phytochemical concentrations, necessitating the establishment of reference metabolite profiles for quality control. The integration of metabolomics-based biomarkers with intelligent bioprocessing strategies holds great promise for enhancing reproducibility and commercial viability.

At the mechanistic level, emerging nutrigenomic evidence suggests that Cili bioactives mediate their beneficial effects partially by modulating the gut–liver axis and lipid metabolism [2,21]. Similarly, Cili vinegar-based formulations further demonstrate efficacy against obesity and dyslipidemia through microbiota modulation [2]. Future research should prioritize clinical validation, dosage optimization, long-term safety assessment, and bioactive stabilization, while advancing circular-utilization strategies for Cili by-products. Together, these efforts will establish Cili as a model for next-generation functional crops.

## 6. Conclusions

This review provides an integrated, multi-omics-driven perspective on Cili (*Rosa roxburghii* Tratt.), synthesizing genomic, transcriptomic, metabolomic, and network pharmacology evidence with mechanistic and translational insights. By linking phytochemical diversity with signaling pathways, functional food processing technologies, and nutraceutical development, this work extends beyond traditional descriptive reviews and establishes a systems-level framework for understanding the biological and industrial potential of this species. Cili has emerged as a model of phytochemical richness and functional diversity within the Rosaceae family, offering exceptional nutritional and therapeutic potential. Advances in metabolomics, transcriptomics, and functional genomics have clarified the molecular basis of its antioxidant, anti-inflammatory, and metabolic regulatory activities. Collectively, these findings demonstrate that the health-promoting effects of Cili arise from synergistic interactions among vitamins, flavonoids, triterpenoids, polysaccharides, and antioxidant enzymes rather than from isolated bioactive molecules.

The integration of multi-omics approaches has not only deepened understanding of Cili metabolic networks but also informed innovations in breeding, processing, and product formulation. As a result, Cili applications now extend beyond traditional products to include functional beverages, nutraceuticals, and bio-based industrial materials, supporting both human health and sustainable economic development.

Looking ahead, the primary challenges remain in standardizing bioactive composition, ensuring product stability, and validating efficacy through rigorously designed clinical and translational studies. Future research should further explore controlled probiotic and mixed-culture fermentation strategies to enhance bioavailability, modulate phytochemical profiles, and support gut health, in line with green processing and functional food development trends. Harnessing systems biology, synthetic biology, and green processing technologies will accelerate the transformation of Cili from a regional specialty fruit into a globally recognized functional crop. Through coordinated scientific and industrial efforts, Cili holds great promise to contribute significantly to future advances in nutrition, preventive medicine, and sustainable resource utilization.

## Figures and Tables

**Table 1:** Summary of Representative Studies on the Biological Effects of Cili (*Rosa roxburghii* Tratt.).

Biological Effect	Study Design/Model	Test Substance (Type and Composition)	Reference Control	Main Outcomes	Statistical Significance	Reference
Antioxidant, cytoprotective	In vitro (HepG2 cells, oxidative stress model)	Whole fruit extract; rich in vitamin C, polyphenols	Untreated control; vitamin C	ROS down; SOD, CAT and GPx up; improved cell viability	*p* < 0.05–0.01	[15]
Antioxidant, anti-inflammatory	In vivo (mice)	Polyphenol-rich pomace extract	Saline; ascorbic acid	MDA down; inflammatory cytokines down; and antioxidant enzymes up	*p* < 0.05	[25]
Anti-inflammatory	In vitro (macrophages, endotoxemia model)	Flavonoid fraction	LPS-treated control	TNF-a and IL-6 up; inhibition of NLRP3 activation	*p* < 0.01	[27]
Hepatoprotective	In vivo (HFD-induced NAFLD mice)	Fermented fruit juice	High-fat diet control	Hepatic lipid accumulation down; antioxidant capacity up	*p* < 0.05	[38]
Antidiabetic, metabolic regulation	In vivo (T2DM mice)	Lactobacillus-fermented juice	Diabetic control	fasting glucose down; insulin sensitivity up	*p* < 0.05	[50]
Gut-liver protection	In vivo (DSS-induced colitis mice)	Whole fruit extract	DSS control; vitamin C	Improved colitis symptoms; superior to vitamin C	*p* < 0.01	[34]
Anti-obesity	In vivo (HFD mice)	Fruit vinegar (polyphenols + organic acids)	HFD control	Body weight gain down and dyslipidemia down	*p* < 0.05	[31]
Anticancer	In vitro (HepG2, DU145 cells)	Polysaccharides; triterpenoids	Untreated cells	Induced apoptosis via ROS/MAPK pathways	*p* < 0.01	[42,43]
Neuroprotective	In vivo (mouse depression model)	Kaji-ichigoside F1	Model control	Neuroinflammation down and BDNF/Akt signalling up	*p* < 0.05	[23]

**Table 2:** Technological Processing, Stabilization, and Functional Food Applications of Cili (*Rosa roxburghii* Tratt.).

Application Type	Processing/Technology	Key Bioactives Retained or Enhanced	Functional Outcome	Stability/Reproducibility Considerations	Reference
Functional beverage	Lactic acid fermentation	Vitamin C, polyphenols, SCFAs	Improved antioxidant and lipid metabolism	Fermentation enhances stability and bioavailability	[2]
Fermented health drink	Mixed microbial fermentation	GABA, polysaccharides, vitamin C	Improved sensory quality and functionality	Controlled strains improve batch consistency	[31]
Nutraceutical extract	Enzyme-assisted extraction	Polysaccharides, flavonoids	Enhanced antioxidant and immunomodulatory effects	Extraction conditions affect molecular integrity	[52]
Pomace utilization	Mono-/mixed-culture fermentation	Residual polyphenols, dietary fiber	Increased antioxidant capacity	Valorizes by-products; reduces waste	[20]
Functional food ingredient	Whole-fruit incorporation	Vitamin C + enzymatic antioxidants	Synergistic antioxidant effects	Whole matrix more stable than purified compounds	[53]
Active food packaging	Composite biopolymer films	Pomace-derived polysaccharides	Extended shelf life; antioxidant protection	Requires standardized raw material profiles	[15]
Industrial biorefinery	Carbonization of pomace	Carbon materials (non-food)	Sodium-ion battery anodes	Circular utilization beyond nutrition	[57]

## Data Availability

No new data were created or analyzed in this study. Data sharing is not applicable to this article.

## Abbreviations

The following abbreviations are used in this manuscript:

SOD	Superoxide dismutase
ROS	Reactive oxygen species
*RrCDF3*	*R. roxburghii* Cycling Dof Factor 3
*RrGGP2*	*Rosa roxburghii* GDP-L-galactose phosphorylase gene
CAT	Catalase
GPx	Glutathione peroxidase
SCFA	Short-chain fatty acid
NO	Nitric oxide
GABA	γ-aminobutyric acid
